# Diagnostic Performance of *Schistosoma* Real-Time PCR in Urine Samples from Kenyan Children Infected with *Schistosoma haematobium*: Day-to-day Variation and Follow-up after Praziquantel Treatment

**DOI:** 10.1371/journal.pntd.0002807

**Published:** 2014-04-17

**Authors:** Natalie V. S. Vinkeles Melchers, Govert J. van Dam, David Shaproski, Anthony I. Kahama, Eric A. T. Brienen, Birgitte J. Vennervald, Lisette van Lieshout

**Affiliations:** 1 Leiden University Medical Centre, Department of Parasitology, Centre of Infectious Diseases, Leiden, The Netherlands; 2 Division of Vector Borne Diseases, Ministry of Health, Nairobi, Kenya; 3 Section for Parasitology and Aquatic Diseases, Faculty of Health and Medical Sciences, University of Copenhagen, Copenhagen, Denmark; Liverpool School of Tropical Medicine, United Kingdom

## Abstract

**Background:**

In an effort to enhance accuracy of diagnosis of *Schistosoma haematobium*, this study explores day-to-day variability and diagnostic performance of real-time PCR for detection and quantification of *Schistosoma* DNA compared to other diagnostic tools in an endemic area before and after treatment.

**Methodology:**

Previously collected urine samples (N = 390) from 114 preselected proven parasitological and/or clinical *S. haematobium* positive Kenyan schoolchildren were analyzed by a *Schistosoma* internal transcribed spacer-based real-time PCR after 14 years of storage. Pre-treatment day-to-day fluctuations of PCR and microscopy over three consecutive days were measured for 24 children using intra-class correlation coefficient. A combined ‘gold standard’ (PCR and/or microscopy positive) was used to measure sensitivity and negative predictive value (NPV) of several diagnostic tools at baseline, two and 18 months post-treatment with praziquantel.

**Principal Findings:**

All 24 repeatedly tested children were PCR-positive over three days with little daily variation in median Ct-values, while 83.3% were found to be egg-positive for *S. haematobium* at day 1 and 75.0% at day 2 and 3 pre-treatment, signifying daily fluctuations in microscopy diagnosis. Of all 114 preselected schoolchildren, repeated microscopic measurements were required to detect 96.5% versus 100% of positive pre-treatment cases by single PCR. At two months post-treatment, microscopy and PCR detected 22.8% versus 69.3% positive children, respectively. Based on the ‘gold standard’, PCR showed high sensitivity (>92%) as compared to >31% sensitivity for microscopy, both pre- and post-treatment.

**Conclusions/Significance:**

Detection and quantification of *Schistosoma* DNA in urine by real-time PCR was shown to be a powerful and specific diagnostic tool for detection of *S. haematobium* infections, with less day-to-day variation and higher sensitivity compared to microscopy. The superior performance of PCR before, and two and 18 months post-treatment provides a compelling argument for PCR as an accurate and reproducible tool for monitoring treatment efficacy.

## Author Summary

*Schistosoma haematobium* is a blood fluke that causes severe urogenital pathology and affects millions of people, mainly in sub-Sahara Africa. Current diagnosis is based on microscopic examination of urine samples, but this method is not only observer dependent, but also known for its low sensitivity and high day-to-day variability. Accurate diagnosis is important to assess community levels of infections for consideration of deworming campaigns, and to monitor treatment efficacy. We evaluated a real-time polymerase chain reaction (PCR) assay for specific detection and quantification of *Schistosoma* DNA in urine samples from 114 preselected *S. haematobium* infected schoolchildren of endemic coastal Kenya and compared the outcome to several other diagnostic methods. Three urine samples collected over three subsequent days from 24 participants were used for Analyzing day-to-day fluctuations in egg counts and *Schistosoma* DNA levels. Urine was also tested two and 18 months after praziquantel treatment. Compared to microscopy, we observed less day-to-day fluctuations and higher sensitivity with real-time PCR, in particular when tested two months after therapy. Real-time PCR is therefore useful for more accurate identification of *S. haematobium*, especially in monitoring control interventions.

## Introduction

An estimated 119 million people are infected with *Schistosoma haematobium* worldwide [1] with sub-Saharan Africa as the major geographical area at risk [2]. In up to 75% of infected individuals both urinary and genital tracts are affected, hence the recent renaming of “urinary” to “urogenital” schistosomiasis. Following effective mass drug administration campaigns, the 65^th^ World Health Assembly held in 2012 realized that intensified control measures were needed to further reduce transmission of schistosomiasis in Africa. The implementation of specific elimination programs were even encouraged [3]. The availability of highly accurate diagnostics is vital for these control programs, particularly in post-treatment situations [4]. In areas with high transmission and subsequently high prevalence and infection intensities, where mass treatment with praziquantel (PZQ) is provided, limited diagnostic sensitivity may be justified as PZQ causes no serious side-effects and generally, the intervention will be cost-effective. However, in moderate and low transmission areas, or in areas with waning post-treatment prevalence, detecting eggs by microscopy becomes more questionable while at the same time cost-effective targeting of mass chemotherapy becomes more essential to avoid the potential emergence of drug resistance [5], [6].

For the diagnosis of urinary tract infections, the ‘gold standard’ remains microscopic detection of ova in urine samples [6]. Nonetheless, parasitological diagnosis has its limitations as the sensitivity is low and may be affected by day-to-day variability in egg excretion [7]. Furthermore, when subjects harbor low worm loads, light infections are often missed by microscopy [8]. Collection of multiple urine samples per individual on consecutive days may increase parasitological sensitivity of microscopy, but is more expensive and also time-consuming [8]. Haematuria represents a good predictor of *S. haematobium* as it correlates well with heavy infection levels [9]. However, blood in urine is a nonspecific symptom of schistosomiasis in areas of low endemicity and can be over- or underestimated depending on the infection prevalence in an area. Haematuria can therefore not be used as direct indicator of infection, but only as a screening method for *S. haematobium* morbidity at the individual level [9].

Alternatively, schistosome parasites release antigenic material into the circulation of their hosts during different stages of their life cycle, thus providing an opportunity to identify these antigens for diagnostic purposes. For example, a circulating soluble egg antigen enzyme-linked immunosorbent assays (cSEA-ELISA) was developed separately for *S. mansoni* [10] and *S. haematobium* [11] for detection of egg antigens. While the *S. mansoni*-specific ELISA could be used as an indicator of tissue egg load [12], urine cSEA levels were found to be primarily a marker of *S. haematobium*-related bladder pathology [11], [13], [14]. In addition, antigens released by the adult worm stage, i.e. circulating cathodic antigens (CCA) and circulating anodic antigens (CAA), can be used to differentiate between active and past infection as they are quickly cleared from the circulation [15], [16]. While a CCA-urine point-of-care (POC) is now commercially available and widely evaluated [17], the CAA assays have recently been much improved [18] with a view towards future POC applications.

In search of diagnostic tools with high overall accuracy when applied to a single sample, the detection and amplification of *Schistosoma* DNA has been evaluated in recent years [19], [20]. Real-time PCR may become an alternative to microscopy and antigen detection for diagnosing *S. haematobium* due to several advantages [19], [20], [21], yet its efficacy is rarely evaluated through population-based surveys in communities originating from schistosomiasis endemic regions. Recent studies based on epidemiological and diagnostic evaluations of real-time PCR using urine samples from communities in Ghana have showed promising results [19], [21]. Nonetheless, there is need to further evaluate the accuracy of real-time PCR for detection of *Schistosoma* DNA in urine samples pre- and post-treatment as limited data are currently available on the performance of real-time PCR as a monitoring tool after chemotherapy [22]. The current study explores pre-treatment day-to-day variability, and diagnostic performance of real-time PCR for detection and quantification of *Schistosoma* DNA compared to microscopy, microhaematuria and cSEA-ELISA in an endemic area before and after treatment.

## Methods

### Ethics Statement

This study was performed retrospectively on urine samples collected by one of the authors (AK) and his team at a larger field survey on human *S. haematobium* in Kenya that was initiated in 1996. All samples were anonymized before analysis. The study was approved by the Ministry of Health's ethical committee (Ministry of Health Kenya) and the Danish Central Medical Ethics Committee. Informed consent was given by the pupils' parents, the education office and the local administration.

### Study Area and Urine Samples

Geographical, demographical and epidemiological details of the study area have been published elsewhere [23]. At collection of data and samples the area was known for high *S. haematobium* prevalence and infection intensity, while *S. mansoni* infections are hardly seen on coastal Kenya [24]. Urines from a total of 114 schoolchildren aged between six and 15 years from two schools (Kibaokiche and Tsunguni) in coastal Kenya were analyzed. The 114 children were part of a larger group selected for post-treatment follow-up studies based on positive *S. haematobium* egg excretion and/or presence of microhaematuria (grade 1) at baseline examination before treatment (attachment S1). The cohort examined for the current study consisted of 114 schoolchildren, who had been present at all follow-up time points and where an urine sample was available for real-time PCR analysis. All infected children in the two schools received supervised treatment with a single regimen of PZQ (40 mg/kg) after baseline investigations and at completion of the study [23]. Aliquots of urine (5–10 ml) were taken before filtration. All samples were stored at −20°C and sent at frozen condition to the Department of Parasitology, Leiden University Medical Centre (LUMC), The Netherlands for quantification of cSEA by ELISA. In 2010, DNA was extracted of 390 urine samples and tested with real-time PCR at the LUMC: i) day-to-day variation was assessed in urine samples collected randomly among 24 schoolchildren prior to treatment on three consecutive days (day  = 1, 2, 3); ii) urine samples from 114 participants were analyzed with the aim of determining the correlation with and performance of PCR as compared to other diagnostic tests at baseline/pre-treatment, two, and 18 months after treatment.

### Parasitological Diagnosis

Parasitological diagnosis was performed in 1996 using standard microscopy on mid-morning urine specimens for day-to-day consistency [11], [14], [23]. Eggs were quantified using a slightly modified Nucleopore syringe urine filtration method, filtering a 10 ml duplicate aliquot from each urine sample [23]. Results were expressed as the number of eggs per 10 ml of urine (eggs/10 ml). Each slide was read by two trained microscopists and discrepancies resolved by consensus before data recording. Eggs exceeding 1000 per 10 ml urine were not counted due to clogging of filters, but recorded as >1000 eggs/10 ml. Egg counts were categorized into no infection (no eggs/10 ml), low (1-<50 eggs/10 ml), and high (≥50 eggs/10 ml) infection intensities. Microhaematuria was assessed using urine-reagent strips Hemastix® (Bayer, UK), according to the manufacturer's instructions. Microhaematuria was categorized into no infection (score 0), “trace” (score 1), low (score 2), moderate (score 3), and high (score 4) infection intensities.

### Circulating Soluble Egg Antigen (cSEA)

The urinary levels of cSEA data were assessed by Kahama *et al.* in 1998, and published previously [11], [13], [14]. In short, a monoclonal antibody-based sandwich ELISA was used for quantification of cSEA in pre-treated urine specimens and concentrations were read against a standard curve. The cut-off for positivity of cSEA samples was determined at 32 ng/ml.

### Real-Time PCR

Urine DNA isolation, amplification and detection were performed as described elsewhere [19], [21]. In short, *Schistosoma* genus-specific primers (Ssp48F and Ssp124R) and the double-labeled probe Ssp78T were used to amplify a 77-bp fragment of the internal transcribed spacer-2 (ITS2) sub-unit. This PCR has been extensively validated on its specificity and appropriate positive and negative controls were included at each PCR run [19], [21]. In addition an internal control (phocin herpes virus 1 (PhHV-1)) was added to each sample reaction for detection of potential inhibition of amplification. A CFX96 real-time PCR detection system (Bio-Rad) was used for amplification and amplicon detection, and CFX Manager version 1.6.514 (Bio-Rad) for related data analysis. The PCR output is expressed as Cyclic-threshold (Ct-) values. Ct-values indicate the number of amplification cycles at which the level of fluorescent signal exceeds the background fluorescence, hence demonstrating parasite-specific DNA loads (infection intensity) in urine samples. The PCR thermocycler was set at 50 cycles in which *Schistosoma* DNA could be amplified. Ct-values were categorized into no infection (Ct = 50), low (35≤Ct≤50), moderate (30≤Ct<35), and high (Ct<30) intensity infections [21].

### Data Analysis

Analysis were performed using SPSS version 20·0 (Statistical Package for the Social Sciences, Chicago, IL, IBM). Main outcome variables were Ct-values and egg counts (continuous). Analysis included: 1) day-to-day variability; 2) correlation between Ct-values and the value of each diagnostic assay; 3) diagnostic performance pre- and post-treatment using sensitivity and negative predictive value (NPV). In case of missing data, outcome measures were calculated based upon available data. Due to limited missing data, this scarcely influenced the results.

#### Day-to-day variability

Pre-treatment egg counts for day-to-day variability were still not normally distributed after log transformation; hence non-parametric statistical methods were used. The median of the three day measurements was calculated per diagnostic assay. To analyze the correlation between each diagnostic parameter, Spearman's correlation was subsequently applied. It calculated the linearity in egg counts and Ct-values as determined by microscopy and real-time PCR. For the analysis of reliability of day-to-day measurements within each diagnostic method (consistency of the diagnostic measure over multiple days), the intra-class correlation coefficient (ICC) was calculated.

#### Correlation of diagnostic assays

For the evaluation of *S. haematobium* infection intensity, concordance between Ct-values as determined by real-time PCR and the value of each diagnostic assay, respectively, was statistically analyzed. As egg counts were skewed even after log transformation, the non-parametric Spearman's Rank Order correlation coefficient (ρ) was used. Cumulative frequencies of positive findings were computed for microscopy by recording additional positive findings of repeated samples. Data were described as total number, interquartile range (IQR), and median value of positive subjects.

#### Estimation of diagnostic accuracy

This study used a combined diagnostic ‘gold standard’ of positive results by real-time PCR and/or positive egg counts as determined by microscopy. A combined ‘gold standard’ is used to create a reliable parasitological measure, which is justified by and described in previous studies [25], [26]. Hence, any infection positive test result, regardless of detection by real-time PCR or microscopy, was considered a true-positive result. This approach is assumed to be a 100% specificity for both tests. Sensitivity and NPV were calculated for each diagnostic tool at all the time points investigated. Diagnostic positivity was coded as a binary variable (infection/no infection). Kappa coefficient (κ) was used to statistically estimate the agreement between one diagnostic technique as compared to another.

## Results

The schoolchildren who provided urine samples for the current study, consisted of 49.1% boys, and ages ranged from six to fifteen years (median: 9.6 years). Kibaokiche school (56.1%) counted 14 more children than Tsunguni school. Prior to treatment, the two schools had similar infection levels. Although schoolgirls had higher egg intensities compared to schoolboys with a median positive value of 128.5 eggs/10 ml (IQR: 21.3, 341.9) for girls and 65.0 (IQR: 15.0, 374.0) eggs/10 ml for boys, boys seemed to be more prone to reinfection (73.2%) and high intensity infection with *S. haematobium* (42.0 eggs/10 ml; IQR: 13.5, 140.0) at 18 months post-treatment than girls (53.4%; 17 eggs/10 ml; IQR: 2.0, 164.0) (χ2: 4.8; p = 0.029). For a more detailed epidemiological description of the results, we refer to the papers by Kahama *et. al.* [13], [14], [23].

### Day-to-Day Variation

The variation in the different diagnostic parameters over three subsequent days prior to treatment of 24 subjects is shown in table 1. Despite that egg and/or microhaematuria positivity was a criterion for study enrolment, four subjects (16.7%) were found egg-negative by microscopy at day 1, and six (25%) on each subsequent day. Median egg counts varied across days, with the lowest median egg count (73 eggs/10 ml) on day 3. Two subjects (8.3%) remained negative by microscopy in all three repeated pre-treatment samples. No new cases were diagnosed on the third day urine sample. Daily variability of microhaematuria was substantial regarding both the proportion of positive and proportion with heavy intensity infections (score 4), the latter ranging from 54.2% to 70.8%. Similarly, daily fluctuations of cSEA-ELISA were considerable between the three subsequent days. All PCR-tested subjects were positive in all three days (100%) while median values and IQRs were comparable.

**Table 1:** Proportion of positive results, interquartile range (IQR), minimum-maximum range, and median per diagnostic test at three different time points (baseline) of 24 *S. haematobium*-positive subjects.

	Day 1	Day 2	Day 3
**Microscopy**			
% positive (N)	83.3% (20)	75.0% (18)	75.0% (18)
IQR	11–346	18–364	17–374
Range	1–1000	2–1000	1–867
Median eggs/10 ml	128	170	73
**Microhaematuria**			
% positive (N)	87.5% (21)	91.7% (22)	79.2% (19)
% with heavy intensity (score 4)	54.2%	62.5%	70.8%
**cSEA-ELISA**			
% positive (N)	87.5% (21)	79.2% (19)	70.8% (17)
IQR	199–3,007	174–2,171	155–1,745
Range	37–18,718	18–7,901	1–4,467
Median ng/ml	689	391	482
**Real-time PCR**			
% positive (N)	100% (24)	100% (24)	100% (24)
IQR	23.7–30.8	22.6–31.0	23.9–32.0
Range	16.4–35.3	21.0–37.2	20.4–37.2
Median Ct-value	26.3	24.4	26.1

All correlation coefficients of the various diagnostic tools using the median values of the three different sampling points pre-treatment (N = 24) were highly significant (p<−0.001). Highest correlation was found between microscopy (eggs/10 ml) and microhaematuria (ρ: 0.81). Correlation with real-time PCR (Ct-values) was similar and best with both cSEA-ELISA (ng/ml) and microhaematuria (ρ: −0.71) as compared to microscopy (ρ: −0.62). Values of real-time PCR are negative as low PCR Ct-values reflect high parasite-specific DNA loads and vice versa. Day-to-day fluctuation of the parameters as determined by ICC was much less for DNA concentrations (R = 0.67; 95%CI: 0.35, 0.85; p = 0.001) than for egg counts (R = 0.29; 95%CI: −0.44, 0.67; p = 0.17). Major day-to-day fluctuations were also found for microhaematuria (R = 0.27; p = 0.19) and cSEA-ELISA (ng/ml) (R = 0.23; p = 0.23).

### Pre- and Post-treatment Positivity and Intensity of *S. haematobium*

**Figure 1:**
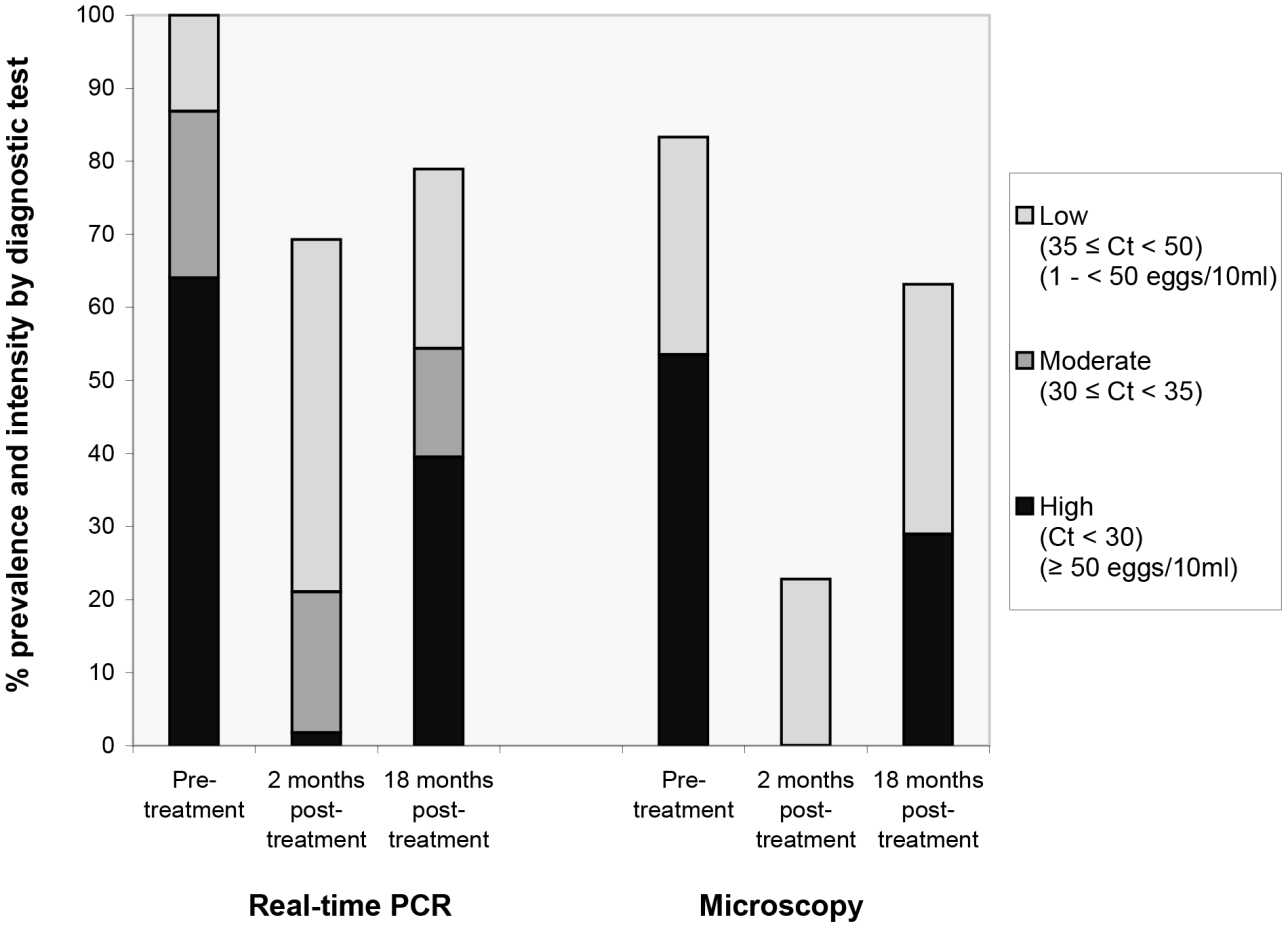
Infection prevalence and intensity group as measured by real-time PCR (Ct-values) and microscopy (egg output) at the different examination time points.

The proportions of *S. haematobium*-positives based on a single sample by the various diagnostic assays are shown in table 2. Only 83.3% of the 114 children were microscopy positive at the first urine samples at baseline. This percentage increased to 96.5% after microscopy of minimum three (up to five) consecutive urine samples, while 100% of the children were PCR-positive based on testing a single urine sample. Treatment with PZQ resulted in significantly reduced intensity of infection after two months, with almost one-fourth (22.8%) of the schoolchildren remaining egg-positive in a single urine specimen and 34.2% in multiple samples. After 18 months, infection levels increased to 63.2% by microscopy of a single urine sample and examination of minimum three (up to five) repeated samples increased positivity to 68.1%. Both microhaematuria and cSEA showed higher proportions of positives at two months post-treatment as compared to microscopy, but both tests missed a substantial number of cases at 18 months post-treatment. Real-time PCR had the highest proportion of *S. haematobium*-positives throughout all time points based on a single sample. A high percentage of positives were ascertained by PCR two months after treatment (69.3%), although DNA levels were relatively low compared to pre-treatment levels (median Ct-value: 35.9). At 18 months post-treatment, positivity increased to 78.9% with a median intensity of 29.9 Ct-values. The course of positive infection and infection intensity at different examination time points is shown graphically in figure 1.

**Table 2:** Cumulative percentages and median (IQR) of positive results for each diagnostic test, based on measurement of single urine samples, at three different examination time points of 114 selected *Schistosomiasis haematobium*-positive schoolchildren.

	Pre-treatment	Post-treatment	Post-treatment
		(2 months)	(18 months)
**Microscopy**			
% positive	83.3%	22.8%	63.2%
Median eggs/10 ml	145.0 (30.0, 578.0)	2.0 (1.0, 7.5)	40.0 (6.5, 156.3)
**Microhaematuria**			
% positive	86.0%	27.2%	45.6%
% with heavy intensity (score 4)	62.3%	0.9%	22.8%
**cSEA-ELISA**			
% positive	79.8%	23.7%*	43.9%
Median ng/ml	551.0 (171.5, 2871.8)	136.0 (64.5, 313.5)	131.0 (51.5, 293.0)
**Real-time PCR**			
% positive	100%	69.3%	78.9%
Median Ct-value	26.6 (23.3, 31.6)	35.9 (34.7, 36.6)	29.9 (24.8, 35.8)

* *Note*: missing data cSEA at two months post-treatment (N = 26).

### Correlation between Diagnostic Values Pre- and Post-treatment

Significant correlations were found between urine DNA levels and egg counts, microhaematuria and cSEA levels, both before treatment as well as 18 months post-treatment (table 3). There was no significant correlation between Ct-values and the other parameters at two months post-treatment.

**Table 3:** Spearman's correlation coefficients between values of each diagnostic tool before treatment, and two and 18 months post-treatment (N = 114).

	Pre-treatment	Post-treatment	Post-treatment
	(Baseline)	(2 months)	(18 months)
**Real-time PCR** (Ct-values)**			
Microscopy (eggs/10 ml)			
Single sample	−0.597*	−0.259	−0.746*
Repeated samples	−0.629*	−0.270	−0.774*
Microhaematuria	−0.576*	−0.116	−0.694*
cSEA-ELISA (ng/ml)	−0.749*	0.044	−0.605*

* *Note*: P-values <0.0001.

^**^ Values of real-time PCR are negative as low PCR Ct-values reflect high parasite-specific DNA loads and vice versa.

### Diagnostic Performance of the Assay Using a Combined ‘Gold Standard’

The performance of each diagnostic tool using a combined ‘gold standard’ (infection positivity by either microscopy or PCR) is shown in table 4. According to the defined ‘gold standard’, 83 samples were positive for *S. haematobium* at two months post-treatment. Microscopy had a sensitivity of 31.3% with a NPV of 35.2%. Kappa agreement between microscopy and the ‘gold standard’ was present (κ: 0.20; p<0.0001). Both microhaematuria and cSEA had similar sensitivities and NPV, and poor Kappa agreements with the ‘gold standard’ (microhaematuria: κ: 0.13; p = 0.036; and, cSEA: κ: 0.03; p = 0.69). Real-time PCR displayed the highest sensitivity and NPV of 95.2% and 88.6%, respectively, at two months post-treatment, as well as high Kappa agreement (κ: 0.92; p<0.0001). PCR missed four out of 83 positive cases.

**Table 4:** Sensitivity and negative predictive value (NPV) at two and 18 months post-treatment.

Test method	Sensitivity (95%CI)	NPV (95%CI)
**Microscopy**		
Post-treatment (2 month)	31% (22, 42)	35% (25, 46)
Post-treatment (18 month)	74% (64, 82)	38% (24, 54)
**Microhaematuria**		
Post-treatment (2 month)	33% (23, 44)	33% (23, 44)
Post-treatment (18 month)	53% (43, 63)	26% (16, 39)
**cSEA-ELISA**		
Post-treatment (2 month)	32% (21, 44)	26% (16, 39)
Post-treatment (18 month)	46% (36, 56)	17% (9, 29)
**Real-time PCR**		
Post-treatment (2 month)	95% (88, 99)	89% (73, 97)
Post-treatment (18 month)	92% (85, 96)	67% (45, 84)

*Note*: Real-time PCR and/or microscopy positivity was used as ‘gold standard.’

At 18 months post-treatment, the sensitivity of microscopy increased to 73.5% but the NPV remained below 40%, while the Kappa-statistic was 0.44 (p<0.0001). Sensitivities of microhaematuria and cSEA also increased at 18 months post-treatment, but to a minor extent compared to microscopy, and both NPVs declined. A total of 98 infections were found using the ‘gold standard’ at 18 months post-treatment, of which 90 cases were detected by real-time PCR thus showing a sensitivity of 91.8%. This was comparable to the sensitivity at two months post-treatment, although the NPV decreased to 67%. The Kappa agreement between PCR and the ‘gold standard’ was 0.76 (p<0.0001).

## Discussion

Analyzing multiple pre-treatment urine samples, we found the proportion of positive cases with eggs, microhaematuria, and/or cSEA to fluctuate significantly on a day-to-day basis, as determined by microscopy, urine-reagent strips, and cSEA-ELISA, respectively. Similarly, daily variations were demonstrated in intensity of infection as determined by microscopy and cSEA-ELISA, and proportion of microhaematuria with score 4. Multiple sample testing can improve the sensitivity of microscopy [27] but also increases workload and costs [8], which is cumbersome in endemic field settings. Previous studies found relatively stable microhaematuria levels over time compared to egg counts [28], [29], but the proportion of microhaematuria-positives and the proportion with score 4 varied considerable in this study. Nevertheless, blood in urine remains a useful marker for preliminary screening of communities to identify those at risk of morbidity [9], [30], [31], as the current study found a strong correlation of microhaematuria with infection as detected by all three diagnostic methods. Furthermore, microhaematuria mirrored egg counts following treatment. Both microhaematuria and cSEA may be useful as infection and pathology markers for *S. haematobium*, as confirmed by relatively good pre-treatment diagnostic sensitivities of both techniques. Alternatively, real-time PCR performed excellently with 100% sensitivity even when using a single urine specimen, with significant correlation and stability over three successive days. The results of the current study are, however, based on a very small sample size, which may have affected the results.

The current study showed that real-time PCR may be a good indicator of infection intensity, as measured by a strong correlation between median Ct-values and egg counts, both pre- and 18 months post-treatment. The correlation between Ct-values and egg counts might be even stronger if egg counts above 1000 eggs/10 ml urine had not been truncated. The correlation with PCR improved when microscopic assessment of infection was based on three to five samples, irrespective of the examination time point. This suggests that real-time PCR could be used as a diagnostic tool providing information about prevalence as well as intensity of infection. Similar significant correlation was found between Ct-values and egg counts by another study, using controls from a non-endemic area, and urine samples known to contain *S. haematobium* eggs as cases [19]. Obeng *et al.* found that the median Ct-values for cases with low-intensity *S. haematobium* infections (≤50 eggs/10 ml urine) was higher than that of cases with intense infections (>50 eggs/10 ml). Discrepancies between these values of microscopy and PCR were likely owing to the lightly infected cases who were excreting very low numbers of eggs, whereby the amount of schistosome genetic material may not reach levels detectable by PCR [19]. There was a poor correlation between egg counts and Ct-values at two months post-treatment. However, it is possible that the linear relation between egg counts and DNA loads may have been disturbed by the temporary effect of treatment [32].

In order to estimate the true-positives of a diagnostic assay in the absence of an accurate and reliable “gold standard”, latent class analysis (LCA) can be employed [33], [34]. However, LCA models are not error-free. The model maintains several assumptions (i.e. conditional independence) that are often violated in practice. Results from at least four different diagnostic assays should be used to account for conditional dependence (i.e. when two assays are based on a similar biological phenomenon), although sufficient results from so many diagnostic assays are not always readily available. In the case of inappropriate use of LCA models, estimations of sensitivity, specificity, and prevalence would only lead to bias [35]. Therefore, analogous to Midzi *et al.* [26] and others [25], [36], this study used the combined results of microscopy and PCR as reference for calculation of diagnostic accuracy based on both methods being 100% specific. Real-time PCR achieved 100% sensitivity after a single test pre-treatment, while microscopy could not detect all positive cases even if more than three independent urine samples were analyzed. Our study shows that the number of positives as determined by PCR at two months post-treatment is considerably higher than revealed by microscopy. Microscopy found a relatively low prevalence after treatment (22.8%) as opposed to 69.3% with PCR. This discrepancy may be explained by the low sensitivity of microscopy (31.3%) in comparison with the ‘gold standard’, which might indicate that PCR-positive, egg-negative cases were false-negatives. PZQ mainly kills mature worms, while immature schistosomes may endure during chemotherapy and redevelop into maturity several weeks after treatment [37]. This temporary discontinue in egg excretion may explain the higher numbers of microscopic false-negatives at two months post-treatment, whereas PCR can still pick-up *Schistosoma*-specific DNA at very low infection intensity levels [38]. Nonetheless, PZQ significantly reduced infection intensity at two months post-treatment as determined by both microscopy and PCR.

Studies have shown that, even with highly effective treatment, many people in endemic regions still harbor sufficient surviving adult schistosomes to account for light but detectable post-treatment egg excretion [5], [39]. In areas of intense transmission, people are likely to have high levels of both patent and pre-patent infections at time of treatment, with PZQ having little effect on immature schistosomes [37]. Considering the lower post-treatment sensitivity of microscopy in this study, the number and intensity of infections may have been underestimated at two months post-treatment. The high number of cases with detectable *Schistosoma* DNA in urine after chemotherapy shows in a more pronounced way that treatment may not be a 100% effective in removing infection [5], [39]. PCR assays detect mostly DNA that originates from schistosomes eggs in urine, stool or organ biopsy samples, and a positive PCR result is therefore dependent on whether the processed sample contains eggs [40]. In chronic, continuously exposed individuals in high intensity infection areas, retention of eggs in the bladder wall may occur [41] or stunted worms may still be present, even after chemotherapy. We therefore cannot rule out with certainty the presence of decaying worms or eggs still expressing parasite DNA [42], although Coulibaly *et al.* found up to 38% higher prevalences with CCA as compared to Kato-Katz in individuals infected with *S. mansoni* post-treatment [36]. As CCA is regurgitated into the bloodstream by actively feeding worms and successive cleared in the host's kidneys [15], [16], Coulibaly *et al.*'s results on *S. mansoni* also indicated the presence of active infection in the host three weeks post-treatment. This suggests that adult worm clearance after chemotherapy may not be as effective as previously thought, and could indicate that real-time PCR may be a useful tool for the evaluation of treatment efficacy in different *Schistosoma* species [8], [43]. Currently, we are awaiting the results of the renewed CCA and CAA-POC, based on the same set of urine samples. Further studies evaluating the *Schistosoma* DNA clearance in relation to circulating antigens after treatment will be of great interest and may provide useful information in relation to a more thorough understanding of post-treatment clearance of worms and eggs. At 18 months post-treatment, infection prevalences returned to 63.2% and 78.9% by microscopy and PCR, respectively, likely caused by reinfection [5], [39]. Given the obvious intense transmission in this area, rapid reinfection with cercariae and hence development of worms is very likely. The consequential increase in number of individuals excreting eggs as well as the quantity of eggs 18 months post-treatment diminishes the random distribution effect of samples without eggs even with active infection well-known in low or moderate infections [7], [8], [43]. Conclusive confirmation of eggs in urine by microscopy is thereby enhanced several months after treatment due to the reoccurrence of high prevalence and infection intensity. In our study, sensitivity of PCR was 92%, and sensitivity of microscopy increased to 74% at 18 months post-treatment.

Considering the high diagnostic accuracy and stability of real-time PCR over multiple time periods, the assay could be a valuable alternative to the current microscopic ‘gold standard’ for diagnosis of schistosome infections. This may especially be true for long-term monitoring of control interventions, as the assay seems particularly useful in demonstrating the presence of low intensity infections in the target population. In addition, we were able to analyze urine samples collected in 1996 and stored at −20°C for almost 15 years with real-time PCR, making retrospective diagnostic and/or epidemiological research feasible. On the other hand, real-time PCR is a laboratory-based test; the required equipment and consumables are expensive and training of laboratory technicians is essential. For cost-effective implementation, this procedure should preferably be performed in a centralized facility, meaning that, for the time being, real-time PCR cannot replace any of the existing methods readily applicable in the field. These include urine-reagent strips to detect haematuria for initial screening of those at risk of morbidity (*S. haematobium*) and urine POC-CCA assays for mapping and rapid screening of at-risk areas for *S. mansoni* [17]. The UCP-CAA strip assay (detecting all schistosome species) is much more sensitive than the POC-CCA. UCP-CAA can be utilized in the current robust format in low-resource settings, but is not yet available as a commercial POC diagnostic assay for schistosome infections [18]. Still, real-time PCR assays can make a valuable contribution in high-tech reference laboratories in especially settings approaching elimination of schistosomiasis, both as a diagnostic and a research tool, helpful in the decision-making process for appropriate intervention delivery, such as in confirmation of successful therapy, and, in the near future, vaccination [38]. Real-time PCR has many advantages: it can be run in a high through-put set-up, results can be standardized, it is possible to quantify parasites, and there is evidently the possibility of targeting several other helminth infections simultaneously in one multiplex real-time PCR. Potentially several non-human *Schistosoma* species could be included in order to study the distribution of zoonotic infections as well. Further studies in different endemic field settings evaluating real-time PCR using recently collected urine samples, as well as studies with larger sample sizes that validates the assay against other diagnostic methods, are encouraged.

## Supporting Information

Checklist S1 STARD checklist.
(DOCX)

Flowchart S1 STARD flowchart.
(TIF)
