# Supplementary figures and images for: Diagnostic Performance of Schistosoma Real-Time PCR in Urine Samples from Kenyan Children Infected with Schistosoma haematobium: Day-to-day Variation and Follow-up after Praziquantel Treatment
Source: PLoS Negl Trop Dis. 2014 Apr 17;8(4):e2807. doi: 10.1371/journal.pntd.0002807 (PMC3990496; doi:10.1371/journal.pntd.0002807)

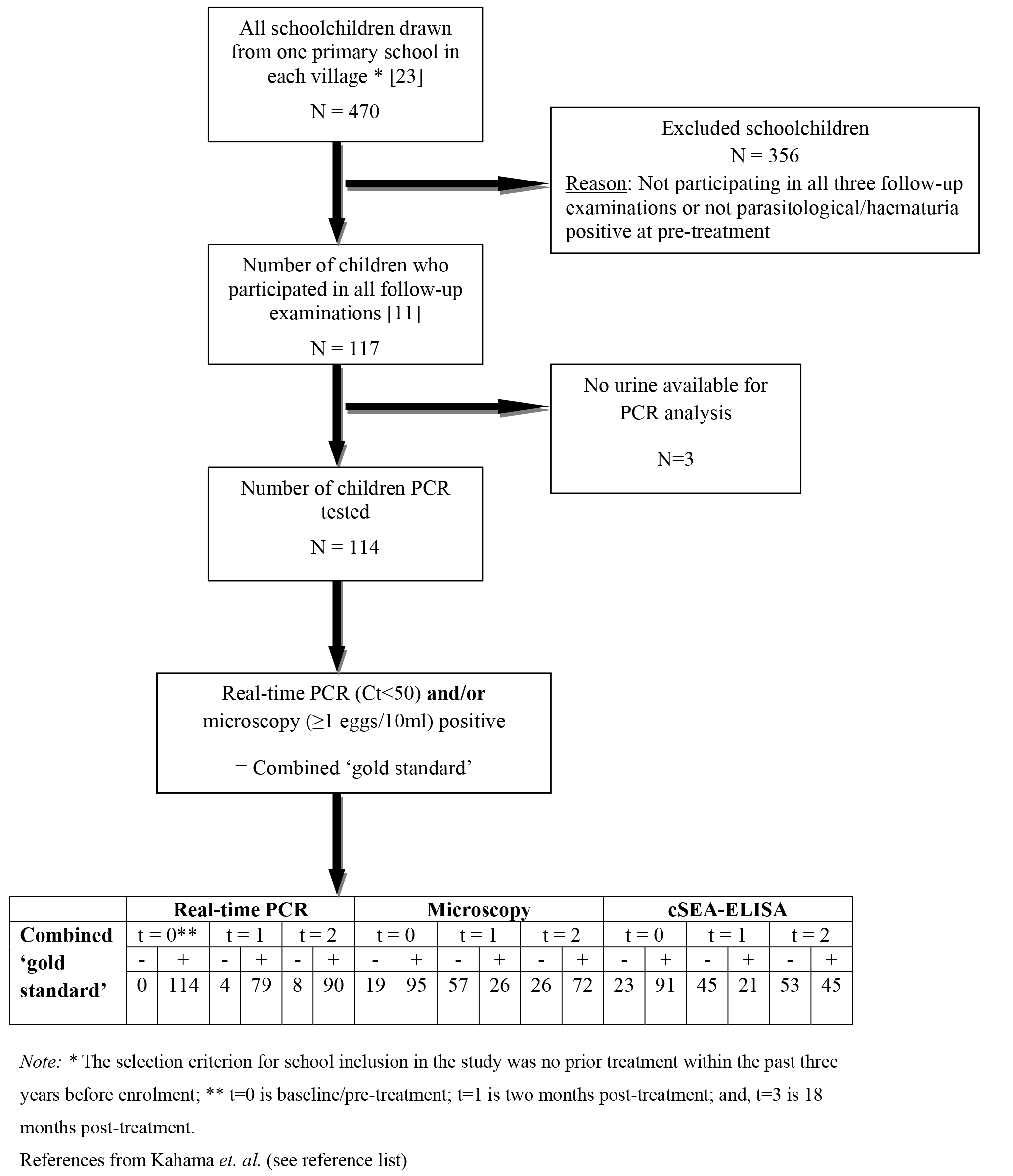

Supplement: Flowchart S1 — STARD flowchart. (TIF) [file pntd.0002807.s002.tif]
